# Association of Dietary Diversity Trajectories With Depressive Symptoms in Chinese Older Adults: Findings From a Nationwide Population-Based Study

**DOI:** 10.1155/da/7630827

**Published:** 2025-10-30

**Authors:** Qianlu Ding, Tingyi Jia, Zhouyang Sun, Yuan Feng, Qianyi Wang, Qianlong Huang, Xiaopeng Sun, Wei Han, Changgui Kou, Wei Bai

**Affiliations:** Department of Epidemiology and Biostatistics, School of Public Health, Jilin University, Changchun, China

**Keywords:** depressive symptoms, dietary diversity score, latent class growth analysis, older adults, trajectory

## Abstract

**Objective:**

Dietary diversity has been found to be related to depressive symptoms. However, the relationship between the trajectory of dietary diversity score (DDS) and depressive symptoms in Chinese older adults remains unclear.

**Methods:**

The longitudinal dataset of Chinese Longitudinal Healthy Longevity Survey (CLHLS) from 2011 to 2018 was used to identify the DDS trajectory among older adults over 65 years old by latent class growth analysis. DDS and depressive symptoms were measured using the food frequency questionnaire and the Center for Epidemiologic Studies Depression Scale-10, respectively. The logistic regression model was used to explore the association between the DDS trajectory and depressive symptoms measured in 2018, and network analysis was used to explore the inter-relationships of depressive symptoms.

**Results:**

A total of 1549 participants were included. This study identified two different DDS trajectories: “persistent high DDS trajectory” and “low but slowly rising DDS trajectory.” After adjusting the covariates, participants with a low but slowly rising DDS trajectory had a higher risk of having depressive symptoms (odds ratio [OR] [95% confidence interval [CI]]: 1.71 [1.34–2.18], *p<*0.001). Comparisons of network structures of depressive symptoms in different DDS trajectories showed that local difference was found in edge CESD2-CESD6 (difficulty with concentrating-feeling nervous/fearful), and the central symptom in both groups was CESD3 “feeling blue/depressed.”

**Conclusion:**

Maintaining high dietary diversity is associated with a lower risk of depressive symptoms among Chinese older adults. Educational campaigns highlighting the importance of dietary diversity could be implemented for this population to lower depression risk and promote healthy aging.

## 1. Introduction

According to the Seventh National Population Census, China's population aged 65 and above has reached 190 million, accounting for 13.5% of the total population [1]. Projections indicate that by 2050, the proportion of older individuals (≥65 years) will rise to approximately 26.9% [2], positioning China among the countries experiencing the most rapid demographic aging globally. With the rapid economic growth, the population faces high-stress lifestyles that may precipitate a substantial increase in mental health issues. Over 100 million Chinese citizens annually experience various types of mental disorders, which collectively constitute more than 20% of the nation's total disease burden, establishing mental health conditions as a major public health concern [3]. Within this context, the health status of older adults demands urgent attention, and the mental health problems of older adults as an important part of health are being paid more and more attention, among which depression is very common [4].

Depression, characterized by persistent low mood manifesting as sadness or emptiness accompanied by cognitive and somatic symptoms, represents a mental disorder that significantly impairs individual functioning [5]. In older adults, sustained depressive affect constitutes the primary clinical manifestation of late-life depression, with depressive symptoms serving as both behavioral expressions of this affective state and critical indicators for depression prediction and diagnosis [6]. Chronic elevation of depressive symptoms to clinical thresholds may culminate in diagnosable depressive disorders [7]. A review indicated that the overall prevalence rate of depressive symptoms among older adults in China was 20.0% [8]. Another study showed that in China, 4.46% of older people suffered from depression, while 35.19% had depressive symptoms [9]. Although the detection rate of depressive symptoms varies according to different periods, populations, and detection tools, on the whole, the prevalence of depressive symptoms among older people in China is constantly rising, which has become a major public health problem that urgently needs to be solved in our country. Depression adversely impacts quality of life, escalates caregiving demands, and elevates suicide risks, ultimately contributing to substantial health and socioeconomic burdens [10]. An integrative review shows that an increasing number of studies have explored risk factors for depression in older adults, including biological, psychological, and social factors [4]. Notably, modifiable lifestyle factors—particularly dietary patterns—have emerged as critical investigational targets [11]. Compared with younger populations, depression in older people demonstrates heightened susceptibility to lifestyle-mediated influences [12]. Since diet can affect some physiological mechanisms of depression, such as inflammation, oxidative stress, brain plasticity, function, and the stress response system, diet may play an important role in the onset and progression of depression [13].

Diet is one of the main determinants of health. The disease burden caused by poor diet quality has increased globally, leading to more than 11 million deaths [14]. In recent years, the modifiable risk factors of overall diet quality have received extensive attention. Compared with other lifestyles, diet is of vital importance in daily life, especially for older people, because it provides a more convenient means of adjustment and optimization [15]. Reviews related to dietary strategies have shown that a diverse, balanced, and nutritious dietary pattern can potentially regulate the nutrient sensing pathways, gut microbiota composition, metabolism, and immunity, thus delaying the aging process [16]. The dietary diversity score (DDS) is considered an effective and clinically relevant measurement index, which reflects nutritional adequacy and diet quality and is used to evaluate the overall diet [17]. Studies have shown that there is a correlation or interaction between DDS and nutritional status [18, 19], suggesting that a higher DDS may indicate nutritional adequacy and improved health status in older people. DDS is associated with the risk of various diseases, for example, a higher DDS is associated with a lower incidence of diabetes [20], metabolic syndrome [21], cardiovascular diseases [22] and all-cause mortality [23]. Currently, many studies have explored an association between DDS and depression. Li et al. [24] found that DDS was negatively correlated with the risk of depressive symptoms in older people.

Network analysis is a method used to assess and visualize the structure and interactions of various symptoms of mental illness, since mental illness arises as a complex network of a series of mutually reinforcing symptoms [25]. In network analysis, each node represents a symptom, and each edge represents the association between two symptoms [26]. The symptoms that have the greatest impact on the whole symptom network are called central symptoms, and clinical interventions targeting central symptoms may lead to more effective outcomes [27]. At present, network analysis has been widely applied to explore the network structure of depressive symptoms in different populations and their inter-relationships with other mental symptoms [28].

However, previous studies have mostly focused on cross-sectional studies and association between dietary diversity and depression using total score, while few studies focused on the inter-relationships between individual depressive symptoms across various dietary diversity trajectory groups. This study aims to explore the dietary diversity trajectory of older adults in China and its association with depressive symptoms, as well as the inter-relationships of depressive symptoms under different trajectories, providing a more realistic and accurate reference for the prevention and treatment of depression among older adults in China.

## 2. Methods

### 2.1. Participants

**Figure 1:**
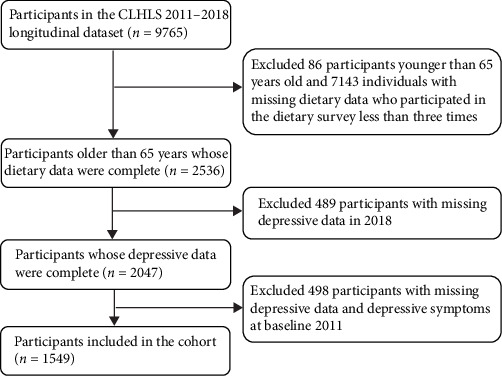
Flowchart of sample selection procedure.

The data of this study was sourced from the Chinese Longitudinal Healthy Longevity Survey (CLHLS) conducted in 23 provinces, municipalities, and autonomous regions across China (1998, 2000, 2002, 2005, 2008, 2011, 2014, and 2018) (source: https://opendata.pku.edu.cn/). The baseline survey of CLHLS initiated in 1998 (wave 1), and the follow-up surveys (wave 2–8) were conducted in 2000, 2002, 2005, 2008, 2011, 2014, and 2018, respectively. CLHLS used multistage stratified random sampling method to survey old people aged 65 years and above. This study used a longitudinal dataset from 2011 to 2018 (wave 6–8). Participants were included if they were 65 years or older, did not have depressive symptoms at baseline, had complete depressive data in wave 8, and had completed dietary-related data in above three waves. Finally, a total of 1549 participants were included in this study, and the flow chart is shown in Figure 1.

### 2.2. Measurements

#### 2.2.1. Dietary Diversity

DDS was used to assess dietary diversity in older adults. CLHLS used a food frequency questionnaire to collect frequency of intake for 13 food groups, including: fruit, vegetables, meat, fish, eggs, food made from beans, salt-preserved vegetables, sugar, garlic, nut products, mushroom or algae, milk products, and tea [29]. If the frequency of fruit and vegetables intake was “almost everyday” or “quite often”, the score was 1, and if it was “occasional” or “rarely” or “never”, the score was 0 [29]. The frequency of the remaining 11 foods included five choices: almost everyday, not everyday but at least once per week, not every week but at least once per month, not every month but occasionally, and rarely or never. If the answer was almost everyday or weekly, it was assigned a value of 1, and the rest was 0. Salt-preserved vegetables and sugar were scored in reverse [30]. Total DDS score ranged from 0 to 13, with a higher score representing a healthier eating pattern.

#### 2.2.2. Depressive Symptoms

CLHLS evaluated depressive symptoms in 2011 using the Phenotypes and eXposures (PhenX) Toolkit [31], which contained two questions. Based on previous studies [31], an individual was defined as having depressive symptoms with an answer “yes” to any of following questions: (1) Have you felt sad, blue, or depressed for 2 weeks or more in last 12 months? (2) Have you lost interest in most things like hobbies, work, or similar activities?

CLHLS evaluated depressive symptoms in 2018 using the Chinese version of Center for Epidemiologic Studies Depression Scale-10 (CESD-10) [32, 33]. CESD-10 consisted of 10 items, and the score of each item ranges from 0 to 3. Three questions were scored in reverse: “I am full of hope for the future,” “I am as happy as I was when I was young,” and “my sleep quality is ideal.” The total CESD-10 score ranges from 0 to 30, with higher scores indicating higher levels of depressive symptoms. A total score of ≥10 was defined as having depressive symptoms [34].

#### 2.2.3. Covariates

In this study, the sociodemographic characteristics, lifestyle, and chronic conditions of the study population were collected as covariates from the follow-up in 2018 (wave 8). Sociodemographic characteristics adjusted in this study included age (years), gender (male or female), residence (urban, town or rural), marital status (married or others), living status (living alone or others), and education level (illiterate, primary school or secondary school and above). Lifestyle includes smoking status (never or others), drinking status (never or others), and exercise (keep exercising or other). Chronic conditions included whether suffering from hypertension and diabetes.

### 2.3. Statistical Analysis

All analyses were performed with R 4.3.3, where *p*  < 0.05 was considered statistically significant. Latent class growth analysis (LCGA) was performed in order to identify different subgroups of individuals with similar patterns of change in DDS measurements at three times points from 2011 to 2018. Models based on two to five trajectories were examined, and the best potential category was determined when the test values of Akaike Information Criteria (AIC), Bayesian Information Criteria (BIC), and sample size adjusted BIC (aBIC) reached relative minimums. The closer the entropy was to 1, the more accurate the classification was. The best model was selected based on the following criteria [35]: (1) Models with lower values of the BIC were preferred. (2) The average posterior probability of each category is greater than 0.7. (3) The proportion of individuals with a high posterior probability (>0.7) in each category exceeds 65%.

Chi-square test and *t*-test were used to test the differences between variables. A random forest method with multiple imputation was used to deal with missing values in the covariates. Four logistic regression models were used to explore the relationship between dietary diversity trajectory and depressive symptoms, and the odds ratios (ORs) and corresponding 95% confidence intervals (CIs) were calculated. Model 1 was unadjusted and model 2 was adjusted for sociodemographic characteristics including age, gender, residence, education level, marital status, and living status. Model 3 was adjusted for covariates in model 2 and lifestyle factors, including smoking, alcohol consumption, and exercise. Model 4 was adjusted for covariates in model 3 and physical conditions, including hypertension and diabetes. All covariates were from follow-up in 2018 (wave 8). Sensitivity analysis was performed to examine the accuracy of the results by deleting individuals with missing values for covariates.

The R software bootnet package (Version 1.6) was used to construct a network analysis model of depressive symptoms in different trajectory groups and examine the stability and accuracy of the network [36]. Additionally, the stability of the centrality index was evaluated using a correlation stability coefficient (CS-C), where CS-C greater than 0.50 was considered perfect and a minimum requirement of CS-C was 0.25 [36]. The R software packages qgraph (Version 1.9.8) were used to depict and calculate the centrality index, and the important central node were determined based on above indices [26, 37]. Differences in network characteristics between different trajectory groups was tested using R-package NetworkComparisonTest (Version 2.2.2) [38].

## 3. Results

### 3.1. Basic Characteristics

A total of 1549 participants were included in the study. As shown in Table 1, the median age of participants was 82 years, and 53.58% (*n* = 830) of participants were male. The proportions of participants who were living in urban areas, married, living alone, and illiterate were 16.33% (*n* = 253), 49.26% (*n* = 763), 19.24% (*n* = 298), and 41.70% (*n* = 646), respectively. About 65.72% (*n* = 1018) of the participants had never smoked, 70.50% (*n* = 1092) of the participants had never drunk, but only 26.47% (*n* = 410) of the participants exercised in the past and present. The percentage of participants with hypertension and diabetes in this study was 39.38% (*n* = 610) and 8.01% (*n* = 124), respectively.

### 3.2. Estimated DDS Trajectory Modeling

**Figure 2:**
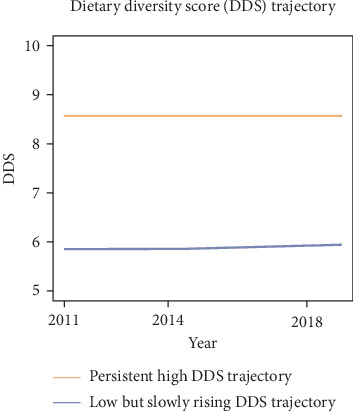
Dietary diversity score trajectories of older people.

As shown in Table S1, the AIC, BIC, aBIC, and entropy results indicated that the model with two trajectory groups had the best fit. The trajectories of DDS were depicted in Figure 2, illustrating two distinct categories of DDS: class 1 was labeled as “persistent high DDS trajectory,” including 482 (31.12%) individuals; class 2 was identified as “low but slowly rising DDS trajectory,” including 1067 (68.88%) individuals.

### 3.3. Association of DDS Trajectories With Depressive Symptoms

**Figure 3:**
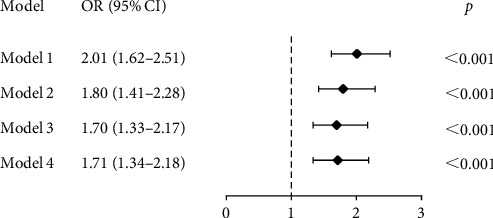
Forest plot of DDS trajectories and depressive symptoms. Note: model 1 was not adjusted; model 2 was adjusted for age, gender, residence, marital status, living status, and education level; model 3 was adjusted for covariates in model 2 and smoking status, drinking status, and exercise; model 4 was adjusted covariates in model 3 +hypertension and diabetes; CI, confidence interval; OR, odds ratio.

Figure 3 shows the associations between different DDS trajectories and the risk of depressive symptoms with persistent high DDS trajectory set as the reference group. Without adjustment (model 1), compared to the reference group, participants with a low but slowly rising DDS trajectory had a higher risk of having depressive symptoms (OR [95% CI]: 2.01 [1.62–2.51], *p*  < 0.001). In adjusted models (model 2, model 3, and model 4), this statistically significant finding was still observed. Typically, after adjusting for sociodemographic characteristics, lifestyle factors, and chronic conditions (model 4), participants in the low but slowly rising DDS trajectory group were more likely to have depressive symptoms compared with the reference group (OR [95% CI]: 1.71 [1.34–2.18], *p<*0.001). Sensitivity analysis was performed by deleting individuals with missing values for covariates, and the association between DDS trajectories and depressive symptoms remained statistically significant (OR [95% CI]: 1.79 [1.37–2.34], *p<*0.001).

### 3.4. Network Analysis

**Figure 4:**
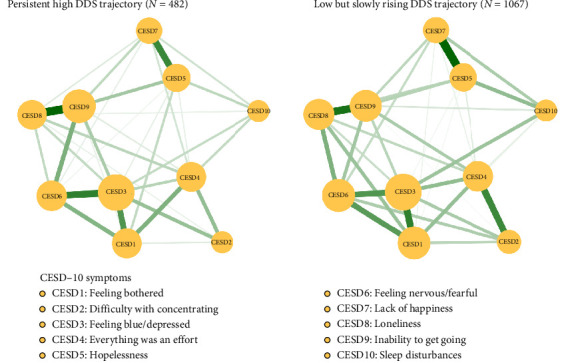
Network structures of depressive symptoms in different trajectories.

Figure 4 shows the network structure of depressive symptom in different DDS trajectories (i.e., persistent high DDS trajectory and low but slowly rising DDS trajectory) among older adults. Comparisons of network structures of depressive symptoms in different DDS trajectories showed that there was no significant difference in network global strength (global strength for persistent high DDS trajectory: 3.571; global strength for low but slowly rising DDS trajectory: 3.777; *S* = 0.205, *p*=0.242), but local difference was found in edge CESD2-CESD6 (difficulty with concentrating-feeling nervous/fearful). Figure S1 shows the strength of each symptom in different DDS trajectories. In the persistent high DDS trajectory, nodes with top three strength values were CESD3 “feeling blue/depressed,” CESD9 “inability to get going,” and CESD6 “feeling nervous/fearful.” In the low but slowly rising DDS trajectory, nodes with top three strength values were CESD3 “feeling blue/depressed,” CESD6 “feeling nervous/fearful,” and CESD1 “feeling bothered.” Regarding network stability, the case dropping bootstrap procedure showed that results were stable (CS-coefficient _persistent high DDS_ = 0.595 and CS-coefficient _low but slowly rising DDS_ = 0.75) (Figure S2). Results of the bootstrap 95% CIs for edges and bootstrapped differences tests for edge weights and node strength are shown in Supporting Information Figures S3–S5.

## 4. Discussion

This study utilized the CLHLS cohort data from 2011 to 2018 to evaluate the impact of long-term dietary diversity trajectories on depressive symptoms in Chinese older people. Two different dietary diversity trajectories (“persistent high DDS trajectory” and “low but slowly rising DDS trajectory”) were analyzed and determined. A total of 482 individuals (31.12%) were classified into the “persistent high DDS trajectory” group. Compared with the “persistent high DDS trajectory” group, the “low but slowly rising DDS trajectory” group observed a higher risk of depressive symptoms, even after adjusting for sociodemographic characteristics, lifestyle factors and chronic conditions.

Similar to previous studies [39, 40], this study employed both logistic regression analysis and network analysis. Logistic regression analysis is used to identify factors associated with an increased risk of depressive symptoms reaching a clinically significant threshold, which may overlook the differences among individual symptoms and their inter-relationships. However, network analysis can be an effective approach to addressing this issue. Depressive symptoms exhibit heterogeneity in terms of their etiology, clinical manifestations, and underlying mechanisms [41], which requires exploration at the symptom level. Interventions or preventive measures targeting central symptoms can usually manage the occurrence and progression of the disease more accurately.

The results showed that the dietary diversity of the Chinese elderly with high DDS was almost unchanged with age, while the dietary diversity of the Chinese elderly with low DDS increased slowly with age. Studies have shown that as people age, the decline in masticatory and digestive functions caused by aging leads to a decrease in dietary diversity among older people [42]. However, due to the rapid economic development and the prosperity of the food market in China at present [43], the dietary diversity of older adults has increased with age in recent years. Elderly people born later can enjoy more benefits and diverse diets brought about by social development, which indicates that the increase in dietary diversity due to socio-economic development can make up for the decline in dietary diversity caused by physiological aging [44]. The DDS of many older people is not high. Therefore, promoting the increase of diverse dietary intake among the population may be an effective strategy to prevent the occurrence of depression.

Diet represents a long-term cumulative process, so it is crucial to explore the impact of continuous and long-term dietary changes or habits on depression. This study had found that maintaining a high level of dietary diversity is associated with a low risk of depression. Previous studies have shown that older people with lower DDS have a higher risk of depressive symptoms [24, 45, 46], which is consistent with the results of this study. Dietary diversity is related to the gut microbiota [47]. There is bidirectional communication between the gut microbiota and the host's central nervous system [48]. This biochemical signaling pathway, also known as the gut-brain axis, is believed to affect mood through neural, metabolic, hormonal, and immune-mediated mechanisms [49, 50]. Long-term dysbiosis of the gut microbiota (GD) can overstimulate the hypothalamic-pituitary-adrenal (HPA) axis and the neuroimmune system, leading to signal transduction dysfunction, inflammation, increased oxidative stress, mitochondrial dysfunction, and neuronal death [51]. Clinically, depressive episodes are associated with dysregulation of the HPA axis [52], and the remission of depressive symptoms is related to the normalization of the HPA axis [53]. Furthermore, GD leads to changes in the blood-brain barrier and intestinal permeability, allowing bacteria and bacterial products to transfer into the systemic circulation [54]. The pro-inflammatory state and low-grade inflammation driven by the intestinal microbiota have been observed in stress-related mental disorders such as depression [55]. This process may be the basis of chronic low-grade inflammation in depression [56].

In the “low but slowly rising DDS trajectory” group, the edge connection between CESD2 “difficulty with concentrating” and CESD6 “feeling nervous/fearful” was stronger than that in the “persistent high DDS trajectory” group. This is in line with the mechanism by which diet affects gut microbiota and thereby influences mood. The experimental study conducted by Ohland et al. [57] proved that a western diet can lead to an increased susceptibility to anxious behaviors and damage memory characteristics. An animal experiment shows that the balance of ω3 to ω6 polyunsaturated fatty acids has an impact on fear memory and synaptic plasticity of the cortico-amygdala [58]. A western diet high in fat and sucrose can damage neurogenesis and reduce the level of BDNF in the hippocampus, adversely affecting cognitive performance [59]. Notably, CESD3 “feeling blue/depressed” had the highest strength and was the central symptom in both groups, consistent with previous network analyses of older adults [28, 60]. Due to physiological degeneration and the increasingly severe diseases related to aging, coupled with the gradual loss of social roles, older people are increasingly prone to symptoms of sadness and depression, further increasing the risk of developing depression [61]. CESD3 “feeling blue/depressed” played a crucial role in the network model of depressive symptoms, so intervention targeting this symptom may have the potential to improve depression.

This study overcame the limitations of static dietary assessment by describing how long-term dietary patterns evolve and predict health outcomes through the LCGA. However, this study had several potential limitations. First of all, although we controlled for some sociodemographic characteristics, lifestyle factors and chronic conditions, due to the observational nature of the study, the possibility of residual confounding factors cannot be ruled out. More importantly, time-dependent confounding factors (e.g., evolving health status between 2011–2018) may affect dietary diversity and depressive symptoms. Future research requires more frequent data waves and advanced causal reasoning methods to better explain these dynamic confounding processes. Second, self-reported dietary diversity and depressive symptoms introduced potential measurement biases. Dietary assessment was conducted through a semi-quantitative food frequency questionnaire, without direct measurement of dietary intake. Despite continuous efforts to improve methods, measurement errors in the assessment of dietary intake in epidemiological studies remained a challenge. These errors were unlikely to be completely eliminated due to factors such as daily changes and self-reporting limitations [62]. Future studies incorporating objective biomarkers can alleviate this problem. Third, since all the participants in this study were from China, the specificity of dietary habits limited the generalization of the research results to other general groups and ethnic groups. Fourth, the instruments used to assess depressive symptoms differed between baseline and follow-up. This may lead to some individuals who had mild depressive symptoms at baseline not being effectively excluded. Fifth, although we constructed the dietary diversity trajectory using three waves of data, the outcome measure (depressive symptoms) was only measured at the end time point in 2018. Therefore, the results of this study reflect the association between trajectory groups and concurrent depressive states, rather than the dynamic longitudinal prediction of the occurrence process of depression by changes in dietary diversity. Future research can set up more intensive follow-up waves, which may be more helpful in clarifying the temporal sequence among variables and testing dynamic causal relationships.

## 5. Conclusion

In conclusion, the results of LCGA showed that individuals with a “low but slowly rising DDS trajectory” were associated with a higher risk of depressive symptoms in the Chinese older adults. CESD3 “feeling blue/depressed” was the central symptom of the depression symptoms network among Chinese older people and may be used as a potential target for intervening in older people at risk or with symptoms of depression. Policies and implementation should focus on increasing dietary diversity among older adults, such as education campaigns on the impact of dietary diversity on the health of older adults, to reduce the risk of depression and promote healthy aging.

## Figures and Tables

**Table 1:** Characteristics of participants in 2018.

Variables	Total (*n* = 1549)	Depressive symptoms		*χ* ^2^/*t*	*p*
		Yes (*n* = 805)	No (*n* = 744)		
Age (years), mean (SD)	83.47 (7.94)	84.47 (8.18)	82.39 (7.53)	−5.19	**<0.001**
Gender, *n* (%)					
Male	830 (53.58)	405 (48.80)	425 (51.20)	6.94	**0.008**
Female	719 (46.42)	400 (55.63)	319 (44.37)	—	—
Residence, *n* (%)					
City	253 (16.33)	110 (43.48)	143 (56.52)	17.1	**<0.001**
Town	558 (36.02)	325 (58.24)	233 (41.76)	—	—
Rural	738 (47.64)	370 (50.14)	368 (49.86)	—	—
Marital status, *n* (%)					
Married	763 (49.26)	336 (44.04)	427 (55.96)	37.28	**<0.001**
Divorced/widowed/never married	786 (50.74)	469 (59.67)	317 (40.33)	—	—
Living alone, *n* (%)					
Yes	298 (19.24)	171 (57.38)	127 (42.62)	4.07	**0.044**
No	1251 (80.76)	634 (50.68)	617 (49.32)	—	—
Educational level, *n* (%)					
0	646 (41.70)	383 (59.29)	263 (40.71)	27.87	**<0.001**
1–6	627 (40.48)	307 (48.96)	320 (51.04)	—	—
>6	276 (17.82)	115 (41.67)	161 (58.33)	—	—
Smoking status, *n* (%)					
Never smoked	1018 (65.72)	567 (55.70)	451 (44.30)	16.11	**<0.001**
Other	531 (34.28)	238 (44.82)	293 (55.18)	—	—
Drinking status, *n* (%)					
Never drank	1092 (70.50)	596 (54.58)	496 (45.42)	9.75	**0.002**
Other	457 (29.50)	209 (45.73)	248 (54.27)	—	—
Exercise status, *n* (%)					
Keep exercising	410 (26.47)	163 (39.76)	247 (60.24)	32.66	**<0.001**
Other	1139 (73.53)	642 (56.37)	497 (43.63)	—	—
Hypertension, *n* (%)					
Yes	610 (39.38)	325 (53.28)	285 (46.72)	0.61	0.436
No	939 (60.62)	480 (51.12)	459 (48.88)	—	—
Diabetes, *n* (%)					
Yes	124 (8.01)	66 (53.23)	58 (46.77)	0.04	0.843
No	1425 (91.99)	739 (51.86)	686 (48.14)	—	—
Dietary diversity score, mean (SD)	6.79 (2.24)	6.32 (2.16)	7.30 (2.22)	8.84	**<0.001**

*Note:* The bold values indicate statistical significance (*p* < 0.05).

Abbreviation: SD, standard deviation.

## Data Availability

This study was carried out using publicly available data from CLHLS at https://opendata.pku.edu.cn/dataverse/CHADS.

## Supporting Information

Supporting Information Table S1 provides fit information for the latent class growth analysis used to identify distinct dietary diversity trajectories, along with sample distribution across different trajectories. Figure S1 visualizes node strength in different network models of depressive symptoms. Figure S2 displays network stability of node strength in different network models of depressive symptoms. Figure S3 shows bootstrapped stability tests for node strength in different network models of depressive symptoms. Figure S4 visualizes bootstrapped stability tests for edge-weights in different network models of depressive symptoms. Figure S5 presents bootstrapped confidence intervals of all edges in different network models of depressive symptoms.
