# Supplementary material for: Association of Dietary Diversity Trajectories With Depressive Symptoms in Chinese Older Adults: Findings From a Nationwide Population-Based Study
Source: Depress Anxiety. 2025 Oct 30;2025:7630827. doi: 10.1155/da/7630827 (PMC12591824; doi:10.1155/da/7630827)
Supplement: Supporting Information — Table S1 provides fit information for the latent class growth analysis used to identify distinct dietary diversity trajectories, along with sample distribution across different trajectories. Figure S1 visualizes node strength in different network models of depressive symptoms. Figure S2 displays network stability of node strength in different network models of depressive symptoms. Figure S3 shows bootstrapped stability tests for node strength in different network models of depressive symptoms. Figure S4 visualizes bootstrapped stability tests for edge-weights in different network models of depressive symptoms. Figure S5 presents bootstrapped confidence intervals of all edges in different network models of depressive symptoms. [file 7630827.f1.docx]

**Association of dietary diversity trajectories with depressive symptoms in Chinese older adults: findings from a nationwide population-based study**

Supplementary Data:

1. Table S1. LCGA fitting information of the dietary diversity trajectories of older people
2. Figure S1. Node strength in different network models of depressive symptoms
3. Figure S2. Network stability of node strength in different network models of depressive symptoms
4. Figure S3. Bootstrapped stability test for ‘node strength’ in different network models of depressive symptoms
5. Figure S4. Bootstrapped stability test for edge-weights in different network models of depressive symptoms
6. Figure S5. Bootstrapped confidence intervals of all edges in different network models of depressive symptoms

Table S1. LCGA fitting information of the dietary diversity score trajectories of older people

| Category | loglik | AIC | BIC | SABIC | entropy | %class1 | %class2 | %class3 | Mean posterior probabilities | Posterior probabilities ＞0.7 |
| --- | --- | --- | --- | --- | --- | --- | --- | --- | --- | --- |
| 1 | -10328.118 | 20662.24 | 20678.27 | 20668.74 | 1.0000000 | 100.000000 |  |  |  |  |
| 2 | -10029.824 | 20071.65 | 20103.72 | 20084.66 | 0.6533724 | 31.116850 | 68.88315 |  | class1: 0.8583  class2: 0.9151 | class1: 80.91%  class2: 89.97% |
| 3 | -9992.996 | 20003.99 | 20052.10 | 20023.51 | 0.5534986 | 17.043254 | 23.24080 | 59.71595 | class1: 0.8594  class2: 0.7424  class3: 0.7766 | class1: 76.14%  class2: 55.28%  class3: 73.95% |

Note: AIC, Akaike Information Criteria; BIC, Bayesian Information Criteria; SABIC, sample size adjusted BIC; LCGA, Latent class growth analysis.


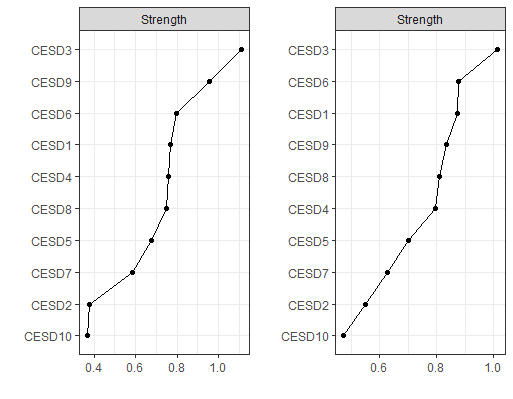


B

A

Figure S1. Node strength in different network models of depressive symptoms (A: persistent high DDS trajectory, B: low but slowly rising DDS trajectory)


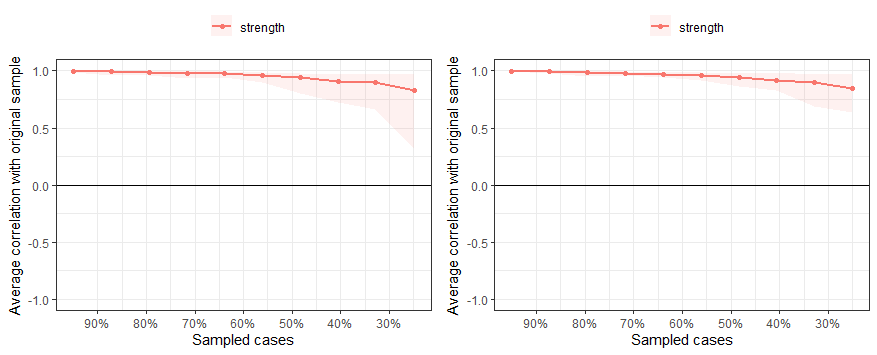


B

A

Figure S2. Network stability of node strength in different network models of depressive symptoms (A: persistent high DDS trajectory, B: low but slowly rising DDS trajectory)


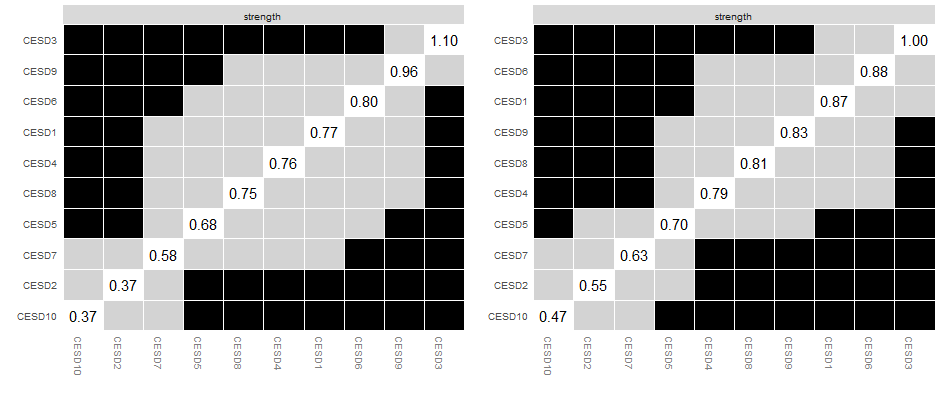


B

A

Figure S3. Bootstrapped stability test for ‘node strength’ in different network models of depressive symptoms (A: persistent high DDS trajectory, B: low but slowly rising DDS trajectory)


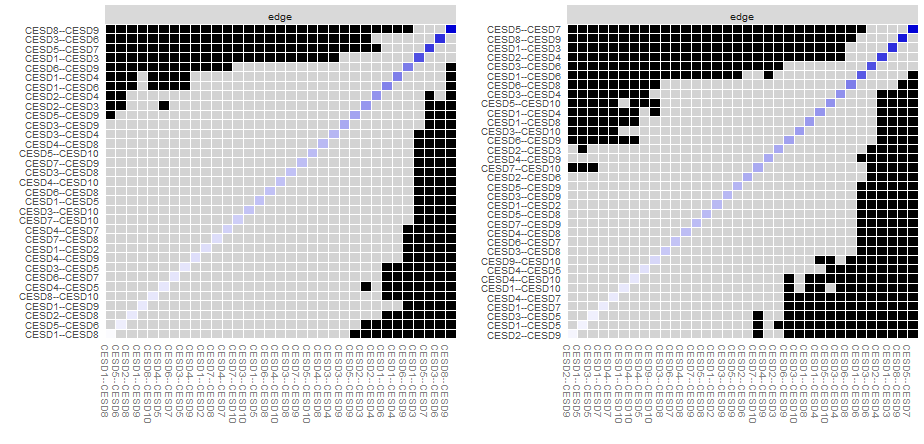


B

A

Figure S4. Bootstrapped stability test for edge-weights in different network models of depressive symptoms (A: persistent high DDS trajectory, B: low but slowly rising DDS trajectory)


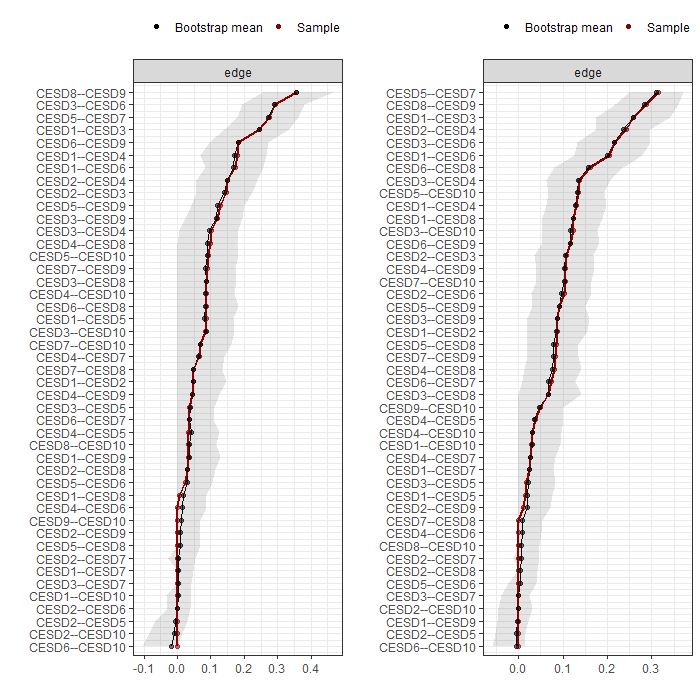


B

A

Figure S5. Bootstrapped confidence intervals of all edges in different network models of depressive symptoms (A: persistent high DDS trajectory, B: low but slowly rising DDS trajectory)
